# Anti-SASP and anti-inflammatory activity of resveratrol, curcumin and β-caryophyllene association on human endothelial and monocytic cells

**DOI:** 10.1007/s10522-021-09915-0

**Published:** 2021-03-11

**Authors:** Giulia Matacchione, Felicia Gurău, Andrea Silvestrini, Mattia Tiboni, Luca Mancini, Debora Valli, Maria Rita Rippo, Rina Recchioni, Fiorella Marcheselli, Oliana Carnevali, Antonio Domenico Procopio, Luca Casettari, Fabiola Olivieri

**Affiliations:** 1 Department of Clinical and Molecular Sciences, DISCLIMO, Università Politecnica delle Marche, Ancona, Italy; 2 Department of Biomolecular Sciences, Università di Urbino “Carlo Bo”, Urbino, Italy; 3 Center of Clinical Pathology and Innovative Therapy, IRCCS INRCA, Ancona, Italy; 4 Department of Life and Environmental Sciences, DiSVA, Università Politecnica delle Marche, 60131 Ancona, Italy

**Keywords:** Nutraceuticals, Polyphenols, Senescence, Inflammaging, SASP

## Abstract

A challenging and promising new branch of aging-related research fields is the identification of natural compounds able to modulate the senescence-associated secretory phenotype (SASP), which characterizes senescent cells and can contribute to fuel the inflammaging. We investigated both the anti-SASP and anti-inflammatory activities of a nutritional supplement, namely Fenoxidol™, composed of turmeric extract bioCurcumin (bCUR), Polydatin (the natural glycosylated precursor of Resveratrol-RSV), and liposomal β-caryophyllene (BCP), in two human cellular models, such as the primary endothelial cell line, HUVECs and the monocytic cell line, THP-1. Replicative and Doxorubicin-induced senescent HUVECs, both chosen as cellular models of SASP, and lipopolysaccharides (LPS)-stimulated THP-1, selected as a model of the inflammatory response, were treated with the three single natural compounds or with a combination of them (MIX). In both senescent HUVEC models, MIX treatment significantly reduced IL-1β and IL-6 expression levels and p16^ink4a^ protein, and also increased SIRT1 protein level, as well as downregulated miR-146a and miR-21 expression, two of the so-called inflamma-miRNAs, more effectively than the single compounds. In THP-1 cells stimulated with LPS, the MIX showed a significant effect in decreasing IL-1β, IL-6, TNF-α, and miR-146a expression levels and Caspase-1 activation, in association with an up-regulation of SIRT1 protein, compared to the single compounds. Overall, our results suggest that the three analysed compounds can have a combined effect in restraining SASP in senescent HUVECs as well as the inflammatory response in LPS-stimulated THP-1 cells.

**Supplementary Information:**

The online version contains supplementary material available at 10.1007/s10522-021-09915-0.

## Introduction

An exciting cutting-edge topic of research concerns the identification of natural compounds that can improve human health. As the population is progressively aging and this phenomenon is accompanied by an increased incidence of age-related diseases (ARDs), the discovery of natural substances able to decelerate the aging process and postpone the development of the most common ARDs has become of widespread interest (Forni et al. 2019). A number of natural compounds capable of interacting with biological processes are contained in food and were therefore named “nutraceuticals” (Biesalski et al. 2009). In recent years, the role of nutraceuticals was extensively investigated in cellular and animal models, as well as in humans, with the aim to prevent and/or contribute to treat ARDs. One of the most interesting hypotheses on the mechanism by which bioactive compounds contained in food could improve health status is their ability to restrain inflammaging (Gurau et al. 2018; Pazoki-Toroudi et al. 2016). Inflammaging is the systemic, low-grade, inflammatory status associated with aging, which is considered a shared risk factor for the most common ARDs (Franceschi 2017; Fulop et al. 2017). The increased burden of senescent cells (SCs) is recognized as key player in promoting inflammaging since SCs are characterized by the acquisition of a senescence-associated secretory phenotype (SASP) with proinflammatory activity (Coppe et al. 2010). SASP, which is fueled by the DNA damage response (DDR), is characterized by NF-kB and NLRP3 inflammasome pathways activation, and by the consequent synthesis and release of a plethora of proinflammatory factors such as interleukins, chemokines, growth factors, matrix-degrading enzymes, reactive oxygen species, and non-coding RNA, i.e. miRNAs (Ishida et al. 2019; Meyer et al. 2017; Munk et al. 2017). Increasing evidence suggests that specific miRNAs, such as miR-21, miR-126, and miR-146a can modulate cellular senescence being involved in the modulation of inflammatory responses and inflammaging (Dimmeler and Nicotera 2013; Harris et al. 2008; O'Connell et al. 2008; Olivieri et al. 2013c; Tili et al. 2007).

Notably, increasing evidence suggests that another main culprit of inflammaging is the repeated stimulation of innate immune responses over time (Prattichizzo et al. 2016). In this framework, both an increased burden of senescent cells during aging and a hyper-stimulation of macrophages over time can be considered key pillars of inflammaging.

Cellular senescence was firstly obtained by long-term culturing of human primary cells (replicative senescence) but can be prematurely triggered in response to several stressors, including oxidative, genotoxic, oncogenic and, therapeutic (induced senescence) (Venturini et al. 2020). In this context, human umbilical endothelial cells (HUVECs) in replicative senescence were used as a well-established in vitro model of SASP and, more recently, Doxorubicin-treated HUVECs were shown to acquire a premature senescent phenotype (Hwang et al. 2020; Venturini et al. 2020). Taking into account that almost all the most common age-related diseases are characterized by endothelial dysfunction, a number of studies on inflammaging were focused on senescent HUVEC models (Hwang et al. 2020; Olivieri et al. 2013a; Wong et al. 2019).

In vivo, the cross-talk between endothelial cells and macrophages is the first step of innate immune activation. Further, endothelial cells can modulate macrophage polarization and function. Monocytic cell line (THP-1) stimulated with LPS can be considered as one of the best-characterized in vitro models of the innate immune pro-inflammatory response (Prattichizzo et al. 2016).

Thus, restraining or delaying SASP acquisition and reducing the increased pro-inflammatory burden of a plethora of antigens over time are emerging as new challenges in aging research (Sabbatinelli et al. 2019; Song et al. 2020).

Several nutraceuticals, including Resveratrol (RSV) and *Curcuma longa,* have been investigated for their potential ability to modulate a number of pathways relevant in the aging process (Howitz et al. 2003; Olivieri et al. 2013c; Schilder et al. 2009; Shishodia 2013). A multitude of data affirmed that the healthful effect on aging is promoted by RSV through the stimulation of sirtuins activities and the inhibition of the inflammatory and stress-related responses (Argyropoulou et al. 2013; Latorre et al. 2017). The biological properties of* Curcuma longa* depend mainly on a yellow-orange lipophilic polyphenolic substance called curcumin family of curcuminoids (McCubrey et al. 2017), which is acquired from the rhizomes of the plant (Shishodia 2013), whereas RSV is a natural phytoalexin found in grapes and red wine (Liu et al. 2017). The age-modulating proprieties of Curcumin has been demonstrated in different animal models, including C.elegans, Drosophila and mice. This compound was found to extent both healthspan and lifespan, mainly suppressing the most relevant proinflammatory pathway NF-kB (Argyropoulou et al. 2013).

BCP and β-caryophyllene oxide (BCPO) are two other naturally occurring phytocannabinoids with bicyclic sesquiterpene structure, present in a large number of plants, that are currently evaluated for their effects on inflammation and pain (Fidyt et al. 2016). BCP can act as an agonist of the peripherally expressed cannabinoid receptor type 2 (CB2), and a number of studies have shown that it is involved in the modulation of inflammatory responses and neuropathic pain, as well as in the modulation of glycaemic and lipidic metabolism (Baldissera et al. 2017; Basha and Sankaranarayanan 2016; Sharma and Kanneganti 2016).

Increasing evidences support the hypothesis of potential additive or synergistic effects of the mixtures compared to single compounds (Iwuchukwu et al. 2011; Masuelli et al. 2014). For example, curcumin synergizes with BCP, exerting an anti-inflammatory activity in human articular chondrocytes indicating the efficacy of the natural compound combination (D'Ascola et al. 2019).

Therefore, we focused our interest on a novel nutraceutical, namely Fenoxidol™ (Mivell S.r.l., Jesi, AN, Italy), composed of bioCurcumin (bCUR), Polydatin and, liposomial β-caryophyllene (BCP). Polydatin is the natural glycosylated precursor of Resveratrol (RSV), characterized by increased bioavailability in humans compared to RSV (Henry-Vitrac et al. 2006).

In this study, we first characterized physicochemically the active ingredients of Fenoxidol™ and subsequently, we evaluated the anti-SASP and anti-inflammatory activity of bCUR, RSV and, BCP, alone or in combination (MIX) as in the Fenoxidol™, on two different human cellular models involved in the fuelling of inflammaging, *i.e.,* endothelial cells and monocytes.

## Materials and methods

### Natural extracts composition

The natural extracts utilized in this study and in the nutritional supplement Fenoxidol™ were analysed in order to characterize their compositions. For the bioCurcumin (bCUR), dissolved in methanol, a high-performance liquid chromatography (HPLC Agilent 1260 Infinity II, Agilent, USA) method was developed to detect at the same time three curcuminoids (curcumin, demethoxycurcumin and bisdemethoxycurcumin) and the main terpene ar-turmerone. The separation was carried with a Zorbax Eclipse XDB-C18, 4.6 × 250 mm, 5 µm column (Agilent, USA) maintained at room temperature. The mobile phase was composed of water containing 0.4% (v/v) acetic acid (A) and acetonitrile (B) with a gradient as follows: 55% A at 0–13 min, 55–44% A at 13–16 min, 44% A at 16–50 min, and 44–0% A at 50–55 min with 10 min of 55% A re-equilibration between individual runs for a total time of 70 min. The injection volume was 20 µL with a constant flow rate of 1 mL/min. The HPLC system was equipped with a diode array detector (DAD) that registered two wavelengths at the same time: 240 nm for the detection of ar-turmerone and 430 nm for the detection of curcuminoids (Chao et al. 2018). Four different calibration curves, one for each evaluated component, were constructed by plotting the mean peak area vs. the concentration of the reference obtaining optimal coefficient of determination (R^2^: 0.9996 for curcumin, 0.9976 for demethoxycurcumin, 0.9997 for bisdemethoxycurcumin and 0.9993 for ar-turmerone) in the concentration range of 0.01–0.1 mg/mL. As for the bioCurcumin, for the evaluation of RSV and polydatin, an HPLC method was utilized. The quantification was carried out in an Agilent Poroshell 120 EC-C18, 100 × 4.6 mm, 2.7 µm column (Agilent, USA) with an isocratic elution composed by 77% of water and 23% of methanol. The injection volume was 20 µL and the flow rate was 1 mL/min. The detection signal was recorded at 306 nm keeping the analysis system at room temperature (Xu et al. 2020). The calibration curve was constructed by dissolving the standard in methanol in a range from 0.01 to 0.1 mg/mL with an R^2^ of 0.9998. The black pepper extract titrated as BCP was provided incapsulated in lyophilized liposomes so, after rehydration in water, the suspension was characterized in terms of average particle size (Z-average) and polydispersity index (PDI) by dynamic light scattering (DLS) using a Malvern Zetasizer Nano S instrument (Malvern Instruments Ltd, UK). The amount of BCP present in the extract was evaluated using a gas chromatograph mass spectrometer (GCMS-QP2010S, Shimadzu, Japan) after a dissolution in acetone. The active molecule was analysed on a Agilent DB-5MS (Agilent Technologies Inc, USA) capillary column (30 m length, 250 µm internal diameter, 0.25 µm film thickness) with the following temperature program: 55 °C for 10 min followed by a temperature rise at a 5 °C/min rate to 220 °C (held for 20 min). Carrier gas was He with a constant flow of 1 mL/min and an injection of 0.2 µL. A calibration curve was constructed from 0.5 to 500 nL/mL with a R^2^ of 0.9995 using BCP standard dissolved in acetone.

### HUVEC and THP-1 culture

HUVECs, primary human umbilical vein endothelial cells, obtained from a pool of donors, were purchased from Clonetics (Lonza, Switzerland) and cultured in endothelial basal medium (EBM-2, CC-3156, Lonza) supplemented with SingleQuot Bullet Kit (CC-4176, Lonza) containing 0.1% human recombinant epidermal growth factor (rh-EGF), 0.04% hydrocortisone, 0.1% vascular endothelial growth factor (VEGF), 0.4% human recombinant fibroblast growth factor (rh-FGF-B), 0.1% insulin-like growth factor-1 with the substitution of arginine for glutamic acid at position 3 (R3- IGF-1), 0.1% ascorbic acid, 0.1% heparin, 0.1% gentamicin and amphotericin-B (GA-1000), and 2% fetal bovine serum (FBS). The cells were seeded at a density of 5000/cm^2^ in T75 flasks (Corning Costar, Sigma Aldrich, St. Louis MO, USA).

Human monocytic THP-1 cells were purchased from ATCC (Rockville, MD, USA) and maintained in RPMI-1640 medium supplemented with 2-mercaptoethanol to a final concentration of 0.05 mM and with 10% heat-inactivated fetal bovine serum, 1% penicillin/streptomycin, and 1% L-glutamine (all from Euroclone, Milano, Italy). The cells were seeded at a density of 2 × 10^5^ cells/ml in T75 flasks.

### Induction and characterization of senescent cells

Replicative senescence (RS) was achieved after a number of replicative passages (measured as population doubling-PD). Population doublings (PDs) were calculated by the formula: ${{\left( {{\text{log}}_{{{1}0}} \left( {\text{F}} \right) - {\text{log}}_{{{1}0}} \left( {\text{I}} \right)} \right)} \mathord{\left/ {\vphantom {{\left( {{\text{log}}_{{{1}0}} \left( {\text{F}} \right) - {\text{log}}_{{{1}0}} \left( {\text{I}} \right)} \right)} {{\text{log}}_{{{1}0}} \left( {2} \right)}}} \right. \kern-\nulldelimiterspace} {{\text{log}}_{{{1}0}} \left( {2} \right)}}$ , where F is the number of cells at the end of the passage and I is the number of seeded cells. Cumulative population doublings (cPD) were calculated as the sum of PD changes. Drug-induced senescence (IS) was obtained by treating HUVECs with Doxorubicin hydrochloride (50 nM) (Sigma Aldrich, Italy) for 24 h. Treated cells were harvested following a 96 h period with fresh medium to acquire the SASP. HUVECs were classified as young or senescent based on cPD as well as on senescence-associated (SA)-β-galactosidase activity and p16^ink4a^ expression. SA-β-Gal activity was detected by using Senescence Detection Kit (BioVision Inc., Milpitas, CA, USA). Briefly, non-confluent, 12-wells plates cultured HUVECs were fixed for 15 min at room temperature, and then washed twice in phosphate-buffered saline (PBS) and incubated overnight at 37 °C with Staining Solution Mix (containing X-Gal). The percentage of β-Galactosidase-positive cells was determined by counting at least 200 cells per well using light microscopy. p16^ink4a^ expression was evaluated by RT-qPCR (Fw: CATAGATGCCGCGGAAGGT; Rv: CTAAGTTTCCCGAGGTTTCTCAGA) and western blot analysis.

### Cell viability assay

MTT (3-(4,5-dimethylthiazol-2-yl)-2,5-diphenyltetrazolium bromide) assay was used to test cell viability. Cells were grown for 24 h in 24-well plates at a density of 5000 cells/cm^2^ before treatments with different doses of the natural compound for 3 h. Briefly, MTT (1 mg/ml) solution was added and incubated for 4 h; the insoluble formazan salt product was solubilized by adding 200 µl of dimethyl sulfoxide (DMSO) and its amount was determined by measuring the optical density at 540 nm using a microplate reader (MPT Reader, Invitrogen, Milano, Italy).

Cell viability was calculated according to the equation (T/C) × 100%, where T and C represent respectively the mean optical density of the treated group and the control group.

### Natural compound treatments

All substances were dissolved in DMSO at a 0.1% final concentration of DMSO in all the solutions. Based on the results of the viability assay, cells were treated with bCUR- 2 µg/ml, RSV- 2 µg/ml and, BCP 20 µg/ml. Considering the total polyphenolic content was 2 µg/ml for all the three tested compounds, we maintained the same amount of polyphenols for the combination treatments, thus the MIX was composed of a total of 2 µg/ml of polyphenols. In addition, a weight ratio equal to 1:1:0.2 (1.6 µg/ml bCUR, 1.6 µg/ml BCP and 0.32 µg/ml RSV) was established to preserve the ratio applied in Fenoxidol™. The treatments with the single compounds and the MIX were carried out for 3 h.

THP-1 cells were treated with 500 ng/ml of LPS (lipopolysaccharide) without or with the single compounds and the MIX for 3 h.

### RNA isolation, mRNA and mature miRNAs expression by RT-qPCR

Total RNA was isolated using the Norgen Biotek Kit (#37500, Thorold, ON, Canada), according to the manufacturer’s instructions. RNA was stored at− 80 °C until use. RNA amount was determined by spectrophotometric quantification with Nanodrop ONE (NanoDrop Technologies, Wilmington, DE, USA). Total RNA (1 µg) was reverse-transcribed using TAKARA Kit (PrimeScript™ RT reagent Kit with gDNA Eraser, Cat: RR047A) based on the manufacturer's instructions. qRT-PCR was performed in a Rotor-Gene Q (Qiagen) using TB Green™ Premix Ex Taq™ (Cat: RR420A) in a 10 µl reaction volume. mRNA quantification was assessed using the 2^−ΔCT^ method. GAPDH and β-actin were used as an endogenous control.

MiRNAs expression was quantified by quantitative real-time PCR (RT-qPCR) using TaqMan miRNA assay (Catalog #4427012—TermoFisher Scientific), according to the manufacturer's protocol. Data were analysed with Rotor Gene Q (Qiagen, Hilden, Germany) with the automatic comparative threshold (Ct) setting for adapting baseline. RT-qPCR data were standardized to RNU44. The 2^−ΔCT^ method was used to determine miRNA expression.

### Western blot analysis

Cell lysates were obtained using RIPA buffer (150 mM NaCl, 10 mM Tris, pH 7.2, 0.1% SDS, 1.0% Triton X-100, 5 mM EDTA, pH 8.0) with protease inhibitor Cocktail (Roche Applied Science, Indianapolis, IN, USA). Protein concentration for each sample was evaluated by Bradford assay. Proteins (30 μg) were analysed by SDS-PAGE, and then transferred to nitrocellulose membrane (Bio-Rad, Hercules, CA, USA). 5% skim milk was used to block the membrane that was then incubated overnight with the primary antibody.

Mouse anti-SIRT1 (Abcam), rabbit anti-Caspase-1 p10 (Santa Cruz Biotechnology), mouse anti-p16^ink4a^ (Santa Cruz) and, rabbit anti-α-tubulin (Cell Signaling), were used as primary antibodies.

Secondary horseradish peroxidase-conjugated anti-mouse and anti-rabbit antibodies were from horse and goat respectively (Vector laboratories, CA, USA). Protein bands were visualized using Clarity ECL chemiluminescence substrate (Bio-Rad) with Uvitec Imager (UVItec,Cambridge,UK) and then quantified using ImageJ software.

Caspase 1 activity was measured as the ratio between band intensity of the pro-Caspasi-1 and of the cleaved Caspasi-1. Each measure was normalized with α-tubulin.

### ELISA assay

Cell culture supernatants were collected at the end of each incubation, centrifuged, and stored at− 20 °C until use. IL-6 ELISA Kit (Cayman chemical, Ann Arbor, USA), IL-1β ELISA Kit (Booster biological technology, Pleasanton, CA) and TNF-α ELISA Kit (Booster biological technology, Pleasanton, CA) were used to measure the concentration of IL-6, IL-1β and TNF-α released in the medium respectively, according to the manufacturer’s instructions.

### Statistical analysis

Summarized data are shown as mean of at least three independent replicates ± SD, SEM or frequency (%). Paired sample T test was used for the analysis of real-time PCR, ELISA and densitometric data. Data analysis was performed using IBM SPSS Statistics for Windows, version 25 (IBM Corp, Armonk, NY, USA). Statistical significance was defined as a two-tailed p-value < 0.05.

## Results

### Natural extracts composition

**Fig. 1:**
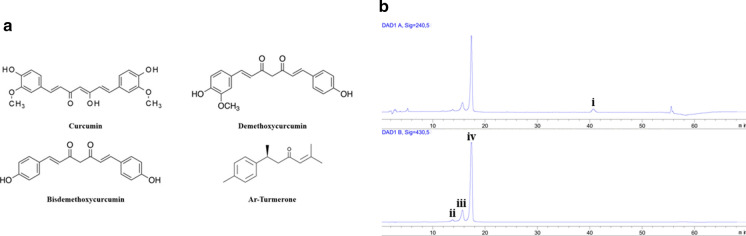
**a** The main molecules of bioCurcumin **b** HPLC–DAD chromatogram with the peaks of ar-turmerone (i) at 240 nm, bisdemethoxycurcumin (ii), demethoxycurcumin (iii) and curcumin (iv) at 430 nm

With the optimized HPLC–DAD technique, the four main molecules of bioCurcumin (bCUR) (Fig. 1a) were detected in a single injection (Fig. 1b) and quantified in 66.3 ± 1.8% of curcumin, 16.4 ± 0.9% of demethoxycurcumin, 8.1 ± 0.3% of bisdemethoxycurcumin and 5.9 ± 0.5% of ar-Turmerone.

Both RSV utilized during the in vitro studies and polydatin present in the nutraceutical formulation was characterized by high purity (> 98%) meanwhile the amount of BCP included in the pro-liposomal formulation contained in Fenoxidol™ was 8.9 ± 0.2%. After resuspension of pro-liposomal powder in water, the main particle size was 204 ± 15 nm with a narrow distribution.

### Resveratrol, β-caryophyllene and bioCurcumin effect on HUVECs and THP-1 cell viability

**Fig. 2:**
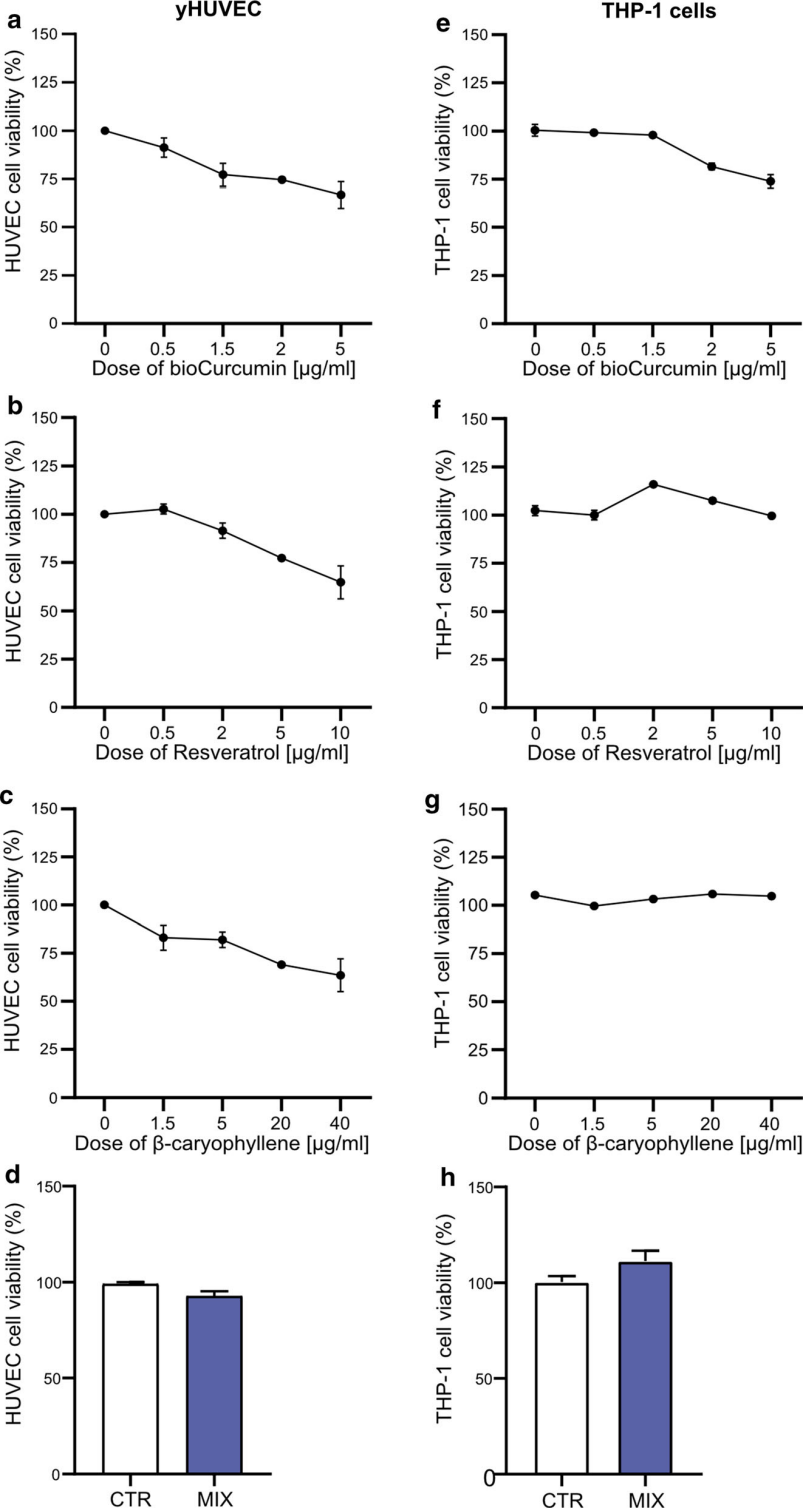
Effect of bCUR, RSV, BCP and MIX on yHUVECs (**a–d**) and THP-1 cells (**e–h**). Cells were treated with different concentrations of bioCurcumin (from 0.5 to 5 µg/ml), Resveratrol (from 0.5 to 10 µg/ml), β‐caryophyllene (from 1.5 to 40 µg/ml) and MIX for 3 h. The viability of cells was determined by MTT assay. The results are expressed as a percentage of cell viability normalized to the viability of DMSO treated cells (CTR) and presented as mean value ± SEM from three independent biological experiments. *bCUR* bioCurcumin, *RSV* resveratrol, *BCP* β‐caryophyllene

RSV, BCP, bCUR and, the MIX cytotoxic effect was evaluated on young HUVECs (yHUVECs) and THP-1 cells (Fig. 2) and on replicative and Doxorubicin-induced senescent HUVEC cells (RS-HUVECs and IS-HUVECs) (Suppl. Figure 1) after 3 h of treatments by MTT assay. Further experiments were performed considering the concentration of compounds that gave approximately 95–75% of viability of treated yHUVECs and THP-1 cells (2 µg/ml bCUR and RSV, 20 µg/ml BCP whereas 1.6 µg/ml bCUR, 1.6 µg/ml BCP, and 0.32 µg/ml RSV were used for the MIX), accordingly to previous reports (Castejon et al. 2017; Faragher et al. 2011; Moghaddam et al. 2019; Zhang et al. 2018).

### Replicative and doxorubicin-induced senescence in HUVECs

**Fig. 3:**
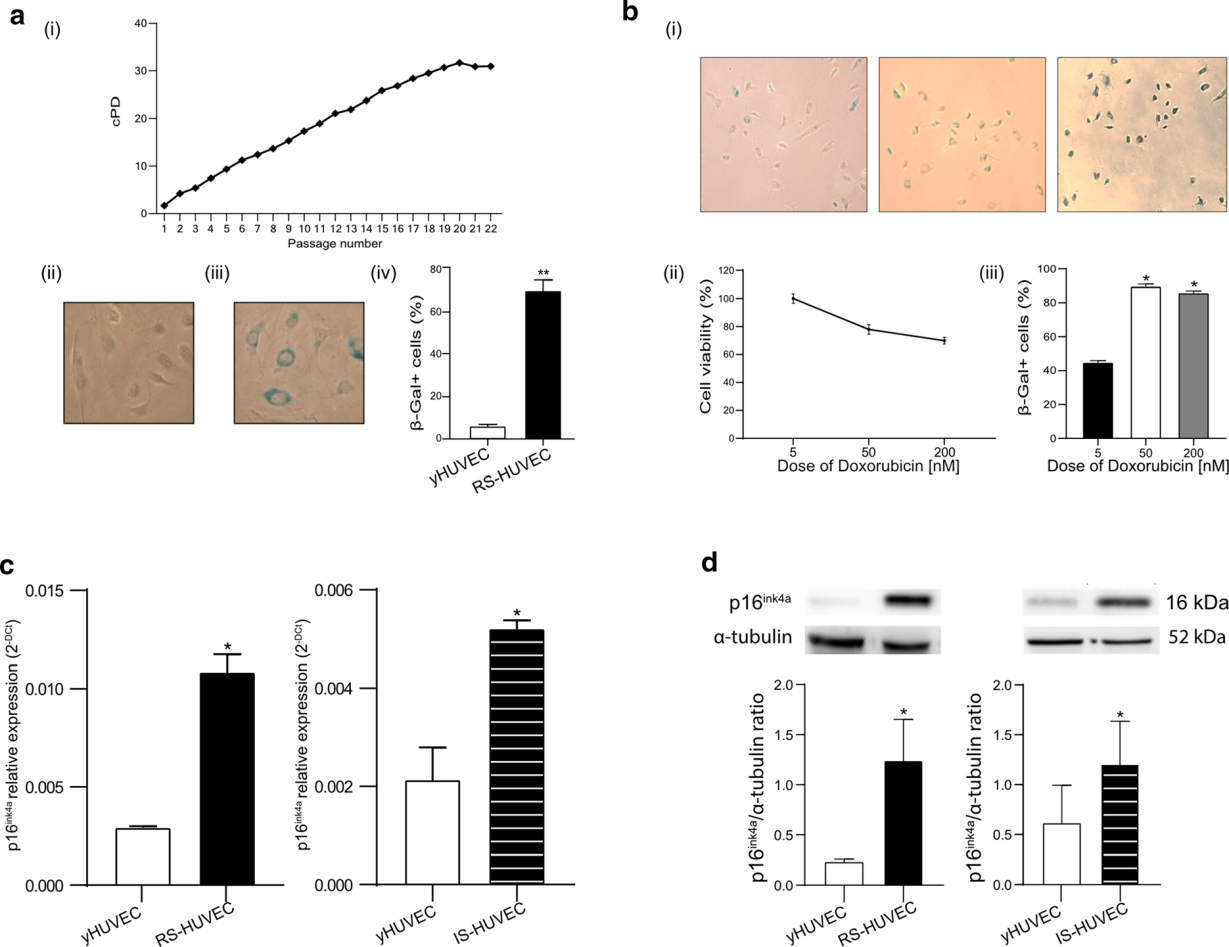
Characterization of endothelial cells in RS- and IS-HUVEC models. **a** Growth curve of a pool of HUVECs—Y-axis: Cumulative Population Doubling (cPD); X-axis: cell passages from P1 to P22 (i); two representative pictures of SA-β-Gal staining of young (ii) and senescent (iii) HUVECs; % of SA- β-Gal (iv). Cells with SA-β-Gal < 10% were considered young cells (yHUVEC), while those with SA- β-Gal > 60% were identified as senescent cells; **b** representative picture of SA- β-Gal-positive cells (i), dose–response curve (ii) and % of SA- β-Gal (iii) after treatment with 5 nM (left image, 50 nM (central image) and 100 nM (right image) of Doxorubicin; **c** relative expression of p16^ink4a^ mRNA in yHUVEC (P4), RS-HUVECs (P21) and IS-HUVECs (Doxorubicin 50 nM); **d** densitometric analysis of p16^ink4a^ protein level in RS-HUVEC and IS-HUVEC. Histograms represent the mean of the protein level and the relative expression measured in three different experiments ± SD. Paired *t* test, *p < 0.05 versus yHUVEC. *RS* replicative senescence, *IS* induced senescence

The effects of natural compounds were analysed in yHUVECs and RS-HUVECs or IS-HUVECs. Senescent status was evaluated by replicative ability, SA- β-Gal activity and p16^ink4a^ expression. yHUVECs were characterized by cPD < 9 and SA-β-Gal positive cells < 5%. RS-HUVECs were defined as cPD > 18 and SA-β-Gal positive cells > 60% (Fig. 3a). To achieve IS-HUVECs model, Doxorubicin cytotoxicity was tested by MTT assay and the concentration of 50 nM corresponded to 80% of cell viability and SA- β-Gal positive cells > 60%, was selected (Fig. 3b). In addition, a significant increase of both p16^ink4a^ mRNA (Fig. 3c) and protein (Fig. 3d) levels, compared to yHUVECs, were selected as cut-off values to establish the senescent status (both RS- and IS-HUVECs).

### Combined natural compounds exert anti-SASP activity on RS- and IS- HUVECs

RS- and IS-HUVECs are both characterized by the acquisition of the senescence-associated secretory phenotype (SASP). Therefore, the effect of natural compounds to restrain the SASP was investigated analysing the expression levels of proinflammatory cytokines, such as IL-1β and IL-6, and some *inflamma-miRs*, like miR-21, -146a and, -126. In addition, three different proteins (i.e., p16^ink4a^, SIRT1 and Caspase-1) involved in the acquisition of SASP were analysed. RS- and IS-HUVECs were treated with the single natural compounds or the MIX. The expression and secretion of IL-6 and IL-1β as well as the expression of miR-146a, miR-126 and miR-21 were significantly increased in RS- and IS-HUVECs compared to yHUVECs.

**Fig. 4:**
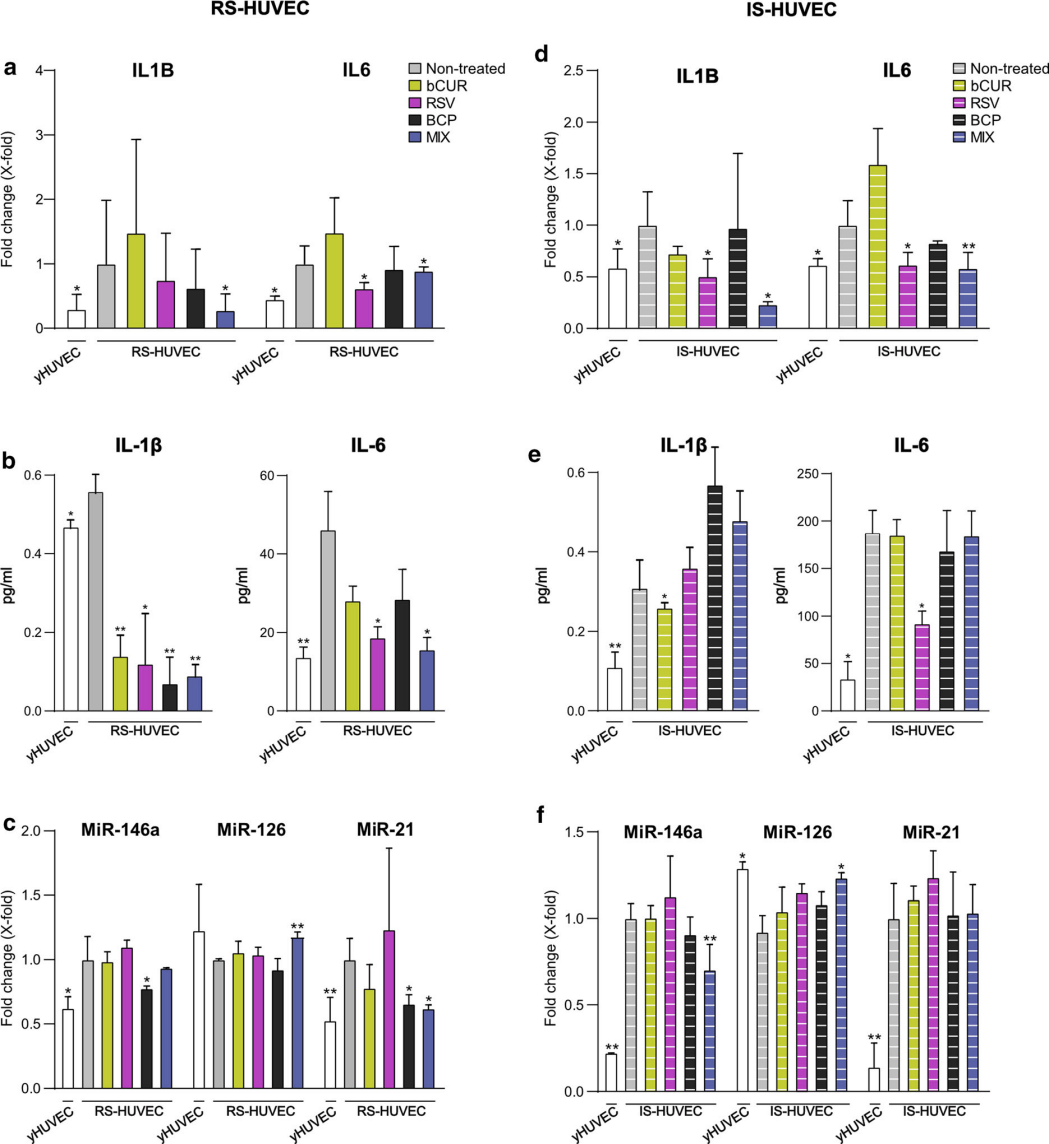
mRNA and miRNA expression, IL-6 and IL-1β concentration after 3 h of treatment with bCUR, RSV and BCP as single compound and in combination in RS- and IS-HUVECs. **a** and **d** mRNA expression of IL-1β and IL-6 in RS- and IS-HUVECs; **b** and **e** concentration (pg/ml) of IL-1β and IL-6 in the culture medium of RS- and IS-HUVECs; **c** and **f** miRNA expression of miR-146a, miR-126 and miR-21 in RS- and IS-HUVEC. Data are reported as fold change *vs* untreated senescence HUVECs according to 2-ΔCt method and using β -actin and RNU48 (respectively for mRNA and miRNA) as housekeeping; histograms represent the mean of the fold change detected in three different experiments ± SD. Paired *t* test, *p < 0.05 versus RS- and IS-HUVECs; **p < 0.01 versus RS- and IS-HUVECs. *bCUR* bioCurcumin, *RSV* resveratrol, *BCP* β‐caryophyllene, *RS* replicative senescence, *IS* induced senescence. RS-HUVECs data are depicted in the left panel (solid-coloured histograms); IS-HUVECs data are reported in the right panel (striped histograms)

The most relevant result was that the treatment of RS- and IS-HUVECs with the MIX was able to significantly decrease IL-1β and IL-6 expression levels in both models of senescence. On the contrary, no single compounds were able to induce the same effects in both RS- and IS- HUVECs. (Fig. 4a and d).

Analysing the levels of IL-1β and IL-6 released in the culture medium, we observed that the MIX was able to induce an important reduction of both cytokines in RS-HUVECs but not in IS-HUVECs (Fig. 4b and e).

**Fig. 5:**
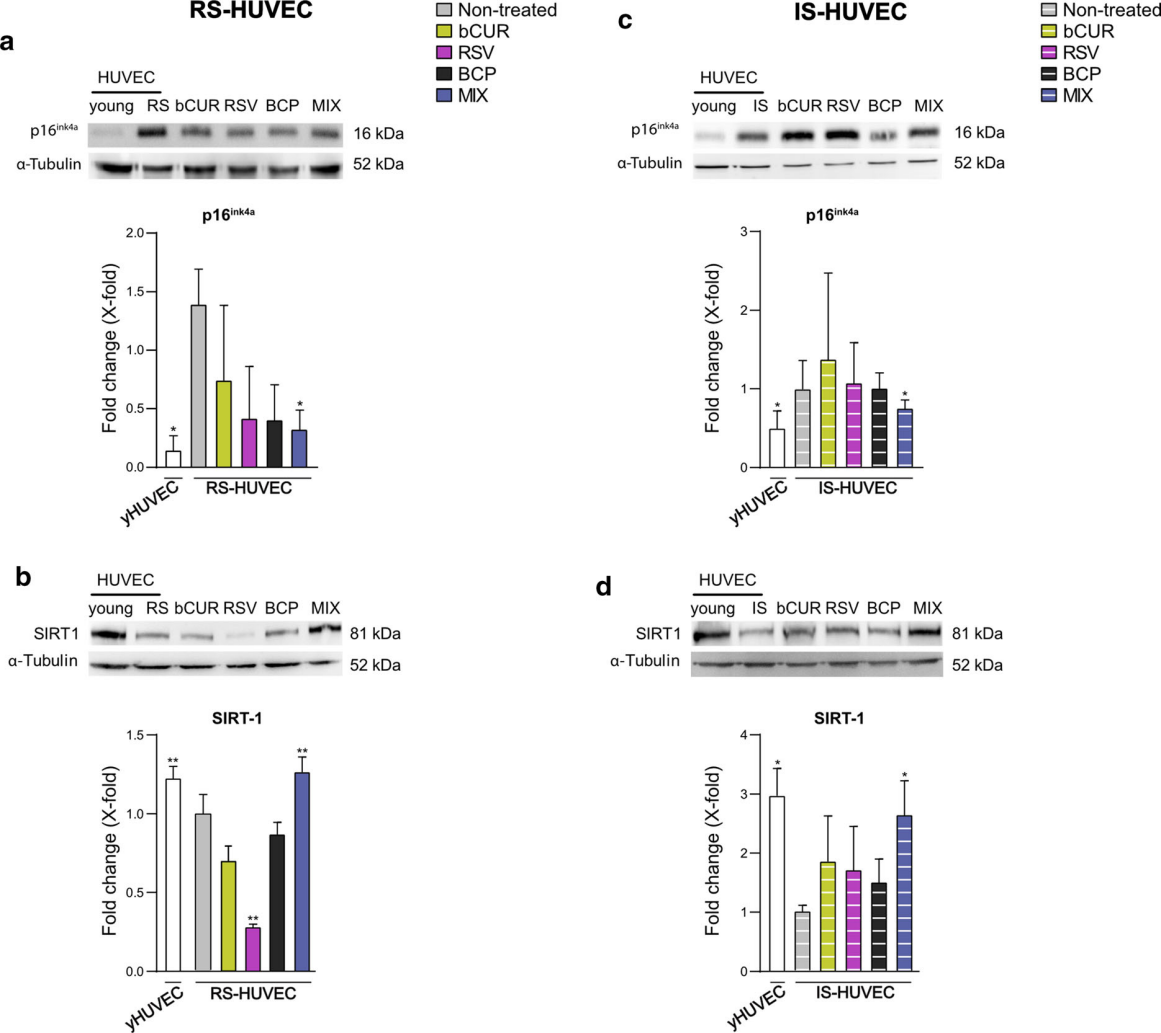
p16^ink4a^ and SIRT1 protein level after treatment with bCUR, RSV and BCP and their combination in RS- and IS-HUVECs. **a** and **c** p16^ink4a^ protein level and densitometric analysis; **b** and **d** SIRT1 protein level and densitometric analysis. Data are reported as fold change vs untreated senescent HUVECs. All data were normalized using α-tubulin as internal control. Bands were quantified by ImageJ; histograms represent the mean of the protein expression detected in three different experiments ± SD. Paired *t* test *p < 0.05 versus RS- and IS-HUVECs; **p < 0.01 versus RS- and IS-HUVECs. bCUR, bioCurcumin; RSV, resveratrol; BCP, β‐caryophyllene; RS, replicative senescence; IS, induced senescence. RS-HUVECs data are depicted in the left panel (solid-coloured histograms); IS-HUVECs data are reported in the right panel (striped histograms)

Moreover, the treatment with the MIX caused a strong down-regulation of miR-21 and miR-146a expression in RS-HUVECs (Fig. 4c) and IS-HUVECs (Fig. 4f) respectively as well as a significant up-regulation of miR-126 level in both senescent HUVEC models (Fig. 4c and f). As regards to protein analysis, as expected, both RS- and –IS-HUVECs showed a significant increase of p16^ink4a^ (Fig. 5a and c) and a reduction of SIRT1 levels (Fig. 5b and d) compared to yHUVECs. Notably the treatment of senescent cells with the MIX downregulated p16^ink4a^ expression in both RS- and IS- HUVECs compared to the non-treated senescent cells (Fig. 5a and c) whilst significantly restored the level of SIRT1 as in yHUVECs (Fig. 5b and d). Furthermore, the pro-Caspase-1 level showed a decreasing trend, which is still not significant, in both RS-and IS-HUVECs treated with the MIX compared to the not-treated senescent HUVECs (Suppl. Figure 2).

### Anti-inflammatory effect of combined natural compounds on THP-1 cells

**Fig. 6:**
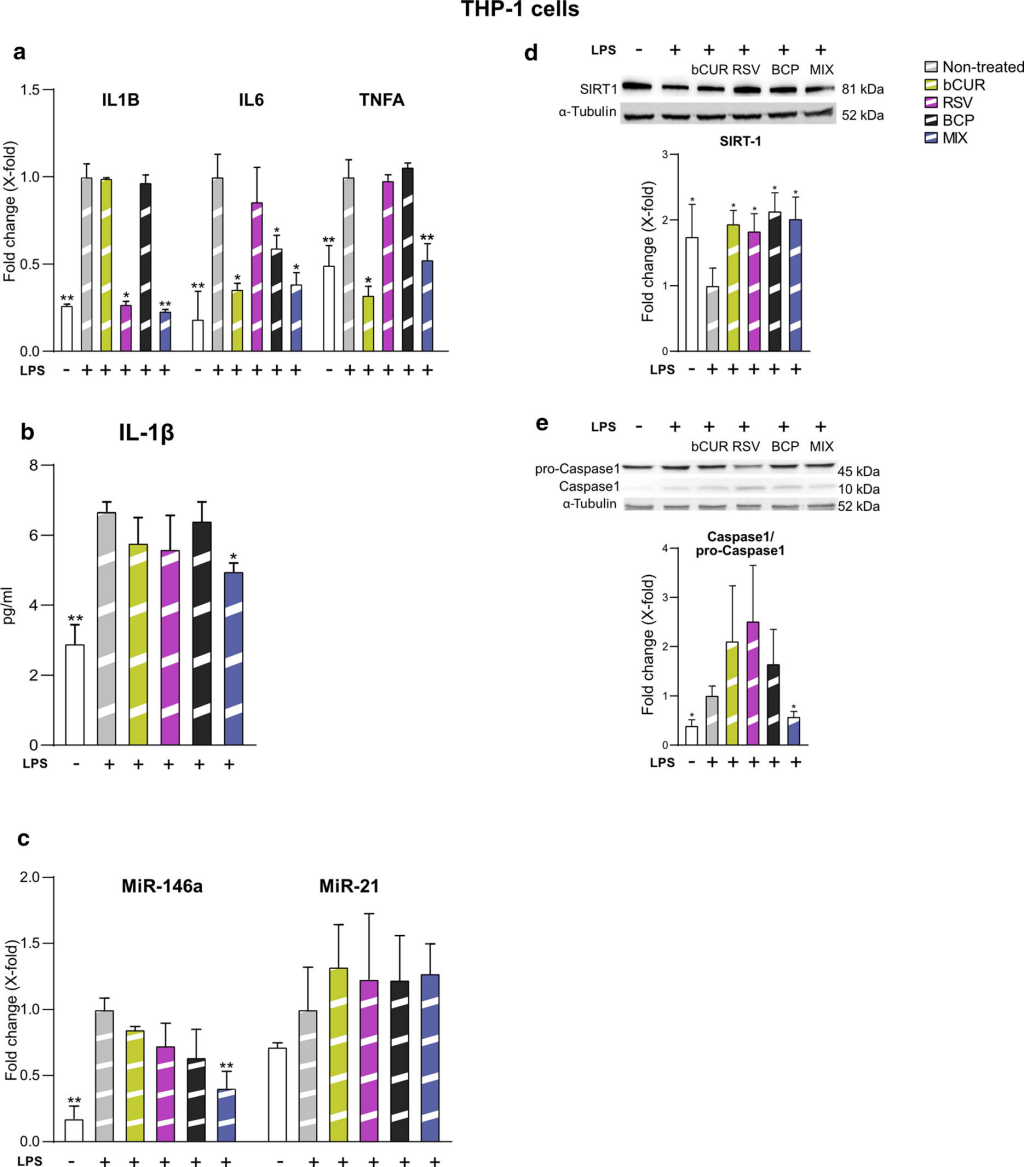
Anti-inflammatory activity of bCUR, RSV and BCP as single compound and in combination in LPS-stimulated THP-1 cells **a** IL-1β, IL-6 and TNFα mRNA expression. Data were reported as fold change vs untreated LPS-stimulated THP1 cells according to 2-ΔCt method and using GAPDH as housekeeping **b** Concentration (pg/ml) of IL-1β in the culture medium of untreated and treated THP-1 cells; **c** miR-146a and miR-21 expression. Data were reported as fold change vs untreated LPS-stimulated THP-1 cells according to 2^−ΔCt^ method and using RNU48 as housekeeping. **d** and **e** SIRT1 and Caspase-1/pro-Caspase-1 protein level and densitometric analysis. Data were normalized using α-tubulin as internal control and reported as fold change versus LPS-stimulated THP-1 cells untreated with natural compounds. Bands were quantified by ImageJ; histograms represent the mean of the protein expression detected in three different experiments ± SD. Paired *t* test, *p < 0.05 versus THP-1 + LPS; **p < 0.01 versus THP-1 + LPS. *LPS* lipopolysaccharide, *bCUR* bioCurcumin, *RSV* resveratrol, *BCP* β‐caryophyllene

The potential anti-inflammatory activity of the natural compounds alone or in combination was also evaluated analysing the expression of IL-1β, IL-6, TNF-α, miR-146a and, miR-21 in LPS-stimulated THP-1 cells. The MIX significantly decreased the expression levels of all tested cytokines (IL-1β, IL-6, TNF -α) (Fig. 6a) and the release of IL-1β in the medium (Fig. 6b) in LPS-stimulated THP-1 cells more efficiently than the single compounds. MiR-146a was found significantly down-regulated by the MIX compared to the single treatments whereas miR-21 was not significantly modulated (Fig. 6c). Regarding the effects on pro-Caspase-1 activation and SIRT1 levels, we observed that the treatment with the MIX was associated with a significant increase of SIRT1 protein level (Fig. 6d) and a significant reduction of pro-Caspase-1 activation (Fig. 6e).

## Discussion

The potential anti-aging function of a number of natural compounds has been extensively investigated in vitro, as well as in pre-clinical tests and clinical trials in humans, suggesting a role either in preventing age-related diseases (ARDs) and in “slowing down” aging itself (Correa et al. 2018; Furst and Zundorf 2014). Cellular senescence is recognized as one of the main triggers of the aging process, since the senescent cells acquired the senescence-associated secretory phenotype (SASP), thus fuelling inflammaging. Several natural compounds have been investigated for their anti-senescence and anti-aging potential effects through the modulation of SASP (Orjalo et al. 2009).

Among the components of SASP, in addition to the well-characterized pro-inflammatory cytokines, there are also some miRNAs, named *inflamma-miRs,* as well as epigenetic-related enzymes, i.e. SIRT1 (Hekmatimoghaddam et al. 2017; Yamakuchi 2012).

Therefore, with the aim to analyse the anti-SASP and the anti-inflammatory effects of some nutraceuticals, we characterized the modulation of a number of SASP related molecules induced by bioCurcumin (bCUR), Polydatin and, β-caryophyllene (BCP), which are the main components of the food supplement Fenoxidol™. The biological properties of the natural compounds were analysed when used individually or combined (MIX) (with the same proportions described in Fenoxidol™) on two different human cellular models, such as HUVECs, in replicative and Doxorubicin-induced senescence, and THP-1 cells stimulated with LPS.

Curcumin and Resveratrol (RSV) have been deeply described for their anti-inflammatory property both in monocytic and endothelial cells (Rana et al. 2016; Schwager et al. 2017; Sun et al. 2015), whereas BCP was only recently identified as an anti-inflammatory compound (Yamaguchi and Levy 2019). However, to the best of our knowledge, this is the first study evaluating the effect in vitro of bCUR, RSV and, BCP combined together (data are summarized in Table 1), as described in the novel dietary supplement Fenoxidol™.

**Table 1:** Summarized data of the anti-SASP and anti-inflammatory effect of bCUR, RSV and BCP alone and in combination (MIX) on RS- HUVECs, IS-HUVECs and THP-1 cells

	RS-HUVEC				IS-HUVEC				THP-1 + LPS				
	bCUR	RSV	BCP	MIX	bCUR	RSV	BCP	MIX		bCUR	RSV	BCP	MIX
IL-1β	ns	ns	ns	*↓	ns	*↓	ns	*↓		ns	*↓	ns	**↓
IL-6	ns	*↓	ns**↓**	*	ns	*↓	ns	**↓		*↓	ns	*↓	*↓
TNF-α	–	–	–	–	–	–	–	–		*↓	ns	ns	**↓
miR-146a	ns	ns	*↓	ns	ns	ns	ns	**↓		ns	ns	ns	**↓
miR-126	ns	ns	ns	**↑	ns	ns	ns	*↑		–	–	–	–
miR-21	ns	ns	*↓	*↓	ns	ns	ns	ns		ns	ns	ns	ns
IL-1β (pg/ml)	**↓	*↓	**↓	**↓	*↓	ns	ns	ns		ns	ns	ns	*↓
IL-6 (pg/ml)	ns	*↓	ns	*↓	ns	*↓	ns	ns		–	–	–	–
SIRT1	ns	**↓	ns	**↑	ns	ns	ns	*↑		*↑	*↑	*↑	*↑
p16^ink4a^	ns	ns	ns	*↓	ns	ns	ns	*↓		–	–	–	–
Caspase-1	ns	*↓	ns	ns	ns	*↓	ns	ns		ns	ns	ns	*↓

*LPS* lipopolysaccharide, *bCUR* bCurcumin, *RSV* resveratrol, *BCP* β‐ caryophyllene, *RS* replicative senescence, *IS* induced senescence, *Ns* not significantly modulated, *down arrow* significantly decreased, *up arrow* significantly increased,—data not present

Student's *t* test *p < 0.05 versus RS- and IS-HUVECs

**p < 0.01 versus RS- and IS-HUVECs; p < 0.05 versus THP-1 + LPS

**p < 0.01 versus THP-1 + LPS

The main result of our study is that the treatment with the MIX promotes significant anti-SASP effects on HUVECs and anti-inflammatory effects on LPS-stimulated THP-1 cells.

In both models of HUVEC senescence, as well as in THP-1, a downregulation of IL-1β and IL-6 mRNA expression was observed. This is a key finding if we consider the primary role of IL-1β in fuelling the inflammatory burden: such cytokine is able to stimulate the expression of several target genes (such as IL-6, MCP-1, IL-8) in different cell types, activating the nuclear factor-kB (Nf-kB) signalling (Weber et al. 2010). Evidence on the anti-inflammatory activity of polyphenols are continuously expanding: Curcumin can decrease several markers of inflammation such as cytokines TNFα and IL-1, the adhesion molecules ICAM-1 and VCAM-1, and some prostaglandins and leukotriens (Shimizu et al. 2019). While it is known that Resveratrol plays anti-inflammatory proprieties which are mainly responsible for its cardioprotective effects (Yahfoufi et al. 2018), it was recently demonstrated that BCP exerts anti-inflammatory activity in high glucose-treated glomerular mesangial cells via the suppression of the NF-κB pathway and the Nrf2 activation (Li et al. 2020).

We observed a significant decreased release of both IL-1β and IL-6 cytokines in the medium of HUVECs in replicative senescence (RS) but not in HUVECs in Doxorubicin-induced senescence (IS). The higher percentage of SA-β-galactosidase (Gal)- positive cells in IS-HUVECs in comparison to RS-HUVECs and the consequent increased amount of cytokines synthesized and released by IS–HUVECs could explain, almost in part, why the three-hour treatment with the MIX is not sufficient to counterbalance the massive release of SASP factors induced by Doxorubicin treatment (Bielak-Zmijewska et al. 2014).

Notably, our study provided evidence that natural compounds exert their biological effects also modulating microRNAs expression (Lin et al. 2017). It has been previously reported that RSV down-regulates some miRNAs involved in the development of cancer (miR-21, miR-30a-5p, miR-19) (McCubrey et al. 2017; Wang et al. 2015). Here we show that in senescent HUVECs the treatment with the MIX was able to reduce the expression of some *inflamma-miRs* (Olivieri et al. 2013b)*,* such as miR-21 and miR-146a and to increase the expression levels of miRNAs involved in the maintenance of endothelial cells functions, *i.e.* miR-126. A significant downregulation of miR-146a was observed in THP-1 cells stimulated with LPS and treated with the MIX. Remarkably, miR-146a and miR-21 can target some proteins belonging to NF-kB pathway, like the tumour necrosis factor receptor-associated factor 6 (TRAF6), IL-1 receptor-associated kinase 1 (IRAK-1) (Taganov et al. 2006), MyD88 (Chen et al. 2013) and programmed cell death protein 4 (PDCD4), respectively (Sheedy et al. 2010). We recently reported that miR-21 loaded on exosomes released by senescent HUVECs can promote features of senescence in younger cells (Mensa et al. 2020). Further, miR-126 plays a pivotal role in modulating vascular development and homeostasis, targeting specific mRNAs such as CXCL12, VCAM-1, SPRED-1 and PIK3R2 (Olivieri et al. 2014). Thus, the significant effect of MIX treatment in increasing miR-126 observed in both senescent HUVEC models strongly suggest its effectiveness in promoting endothelial cell function. In addition the treatment with the MIX was associated with increased SIRT1 levels in senescent HUVECs and with decreased pro-Caspase-1 activation in inflamed THP-1, which are molecules involved in inflammation, aging and ARDs (Cordero et al. 2018). SIRT1 is the most studied sirtuin in mammals, with many roles in several tissues and organs, including the ability to restrain SASP at the transcriptional level (Hayakawa et al. 2015); it also regulates the cellular response to stressors and delays vascular senescence by activating eNOS and suppressing the p53 and NF-kB pathways (Kida and Goligorsky 2016). Notably, SIRT1 blocks the monocyte transmigration into the arterial wall, playing anti-inflammatory effects in endothelial cells (Kida and Goligorsky 2016). Interestingly, increasing evidence suggests that a number of dietary intake of RSV, catechins, EGCG, propolis extracts, creosol, and luteoloside may promote health and extend the lifespan via multiple mechanisms, including also the suppression of NLRP3 activation (Chuang et al. 2014). The NLRP3 inflammasome is essential to mediate the immune responses through the activation of caspase-1 and IL-1β. Indeed, the inflammasome association prompts a proteolytic cleavage of dormant pro-Caspase-1 into active Caspase-1, which converts the cytokine precursors pro-IL-1β into mature and biologically active IL-1β (He et al. 2016).

We observed an increased IL-1β synthesis and release by senescent HUVECs and by LPS-stimulated THP-1 cells. Importantly, the treatment with the MIX was associated with a significant reduction of IL-1β transcription levels in all analysed cellular models, suggesting the ability of MIX to restrain the pro-inflammatory responses and SASP.

Interestingly, we also observed a slight but significant reduction of p16^ink4a^ protein level, a well-known regulator of cellular senescence (Campisi 2011), in both RS-and IS-HUVEC treated with the MIX for 3 h, suggesting a possible anti-aging effect of the three natural compound combination. Notably, our results on HUVECs are in accordance with data suggesting that quercetagetin 3,4′-dimethyl ether exerts anti-senescence effects, confirmed by the modulation of p53 and p21 protein levels, in RS-HUVECs (Yang et al. 2015).

Overall, our results suggest that the MIX of bCUR, RSV and, BCP exerts a combined anti-inflammatory/anti-SASP activity in THP-1 cells and RS- and IS- HUVECs. However, additional studies are needed to confirm the effect of the MIX analysing a larger panel of cell lines (e.g., HAEC, HCAEC). Moreover, further studies on miR-126 target genes (e.g., CXCL12, VCAM-1, SPRED-1 and PIK3R2), could clarify the effects of the MIX on endothelial homeostasis.

Considering that endothelial and innate immune cells are key players in activating and perpetuating the inflammatory responses that fuel inflammaging, which is a common risk factor for the development of the most common ARDs, our data reinforce the hypothesis that specific combinations of nutraceuticals could be more effective than single compounds in promoting healthy aging.

## Supplementary Information

Below is the link to the electronic supplementary material.

Supplementary file1 (DOCX 434 kb)

## Data Availability

All data generated or analyzed during this study are included in this published article.
