# Supplementary material for: Anti-SASP and anti-inflammatory activity of resveratrol, curcumin and β-caryophyllene association on human endothelial and monocytic cells
Source: Biogerontology. 2021 Mar 11;22(3):297–313. doi: 10.1007/s10522-021-09915-0 (PMC8084815; doi:10.1007/s10522-021-09915-0)
Supplement: Supplementary file 1 — Supplementary file1 (DOCX 434 kb) [file 10522_2021_9915_MOESM1_ESM.docx]

**SUPPLEMENTARY INFORMATION**

**Anti-SASP and anti-inflammatory activity of Resveratrol, Curcumin and β-caryophyllene association on human endothelial and monocytic cells**

Giulia Matacchione^1^°, Felicia Gurău^1^°, Andrea Silvestrini^1^*, Mattia Tiboni^2^*, Luca Mancini^2^, Debora Valli^1^, Maria Rita Rippo^1^, Rina Recchioni^3^, Fiorella Marcheselli^3^, Oliana Carnevali^4^, Antonio Domenico Procopio^1,3^, Luca Casettari^2^, Fabiola Olivieri^1,3^.

1 Department of Clinical and Molecular Sciences, DISCLIMO, Università Politecnica delle Marche, Ancona, Italy

2 Department of Biomolecular Science, Università di Urbino "Carlo Bo", Urbino, Italy

3 Center of Clinical Pathology and Innovative Therapy, IRCCS INRCA, Ancona, Italy

4 Department of Life and Environmental Sciences, DiSVA, Università Politecnica delle Marche, 60131 Ancona, Italy

° These authors equally contributed to the manuscript

*Correspondence: Andrea Silvestrini: a.silvestrini@pm.univpm.it ; Tel.: +39.0712206243

+ Mattia Tiboni: mattia.tiboni@uniurb.it Tel: +39.0722303338


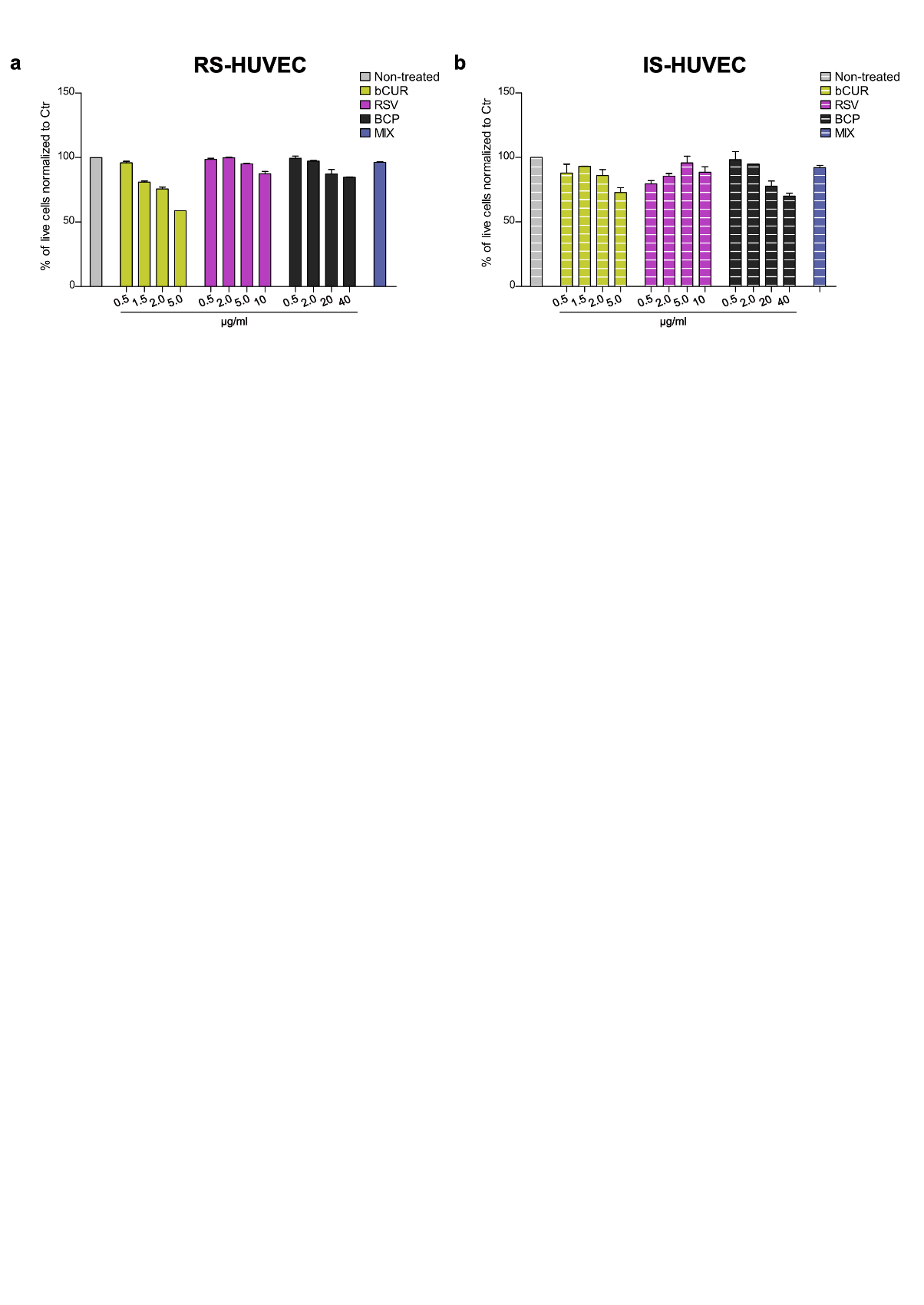


**Supplementary Figure 1 Effect of bCUR, RSV, BCP and MIX on RS-HUVECs (a) and IS-HUVECs (b)**. Cells were treated with different concentrations of bioCurcumin (from 0.5 µg/ml to 5 µg/ml), Resveratrol (from 0.5 µg/ml to 10 µg/ml), β‐caryophyllene (from 1.5 µg/ml to 40 µg/ml) and MIX for 3h. The viability of cells was determined by MTT assay. The results are expressed as a percentage of cell viability normalized to the viability of DMSO treated cells (Non-treated) and presented as mean value ± SEM from three independent biological experiments. bCUR, bioCurcumin; RSV, Resveratrol; BCP, β‐caryophyllene. RS-HUVECs data are depicted in the left panel (solid-coloured histograms); IS-HUVECs data are reported in the right panel (striped histograms).


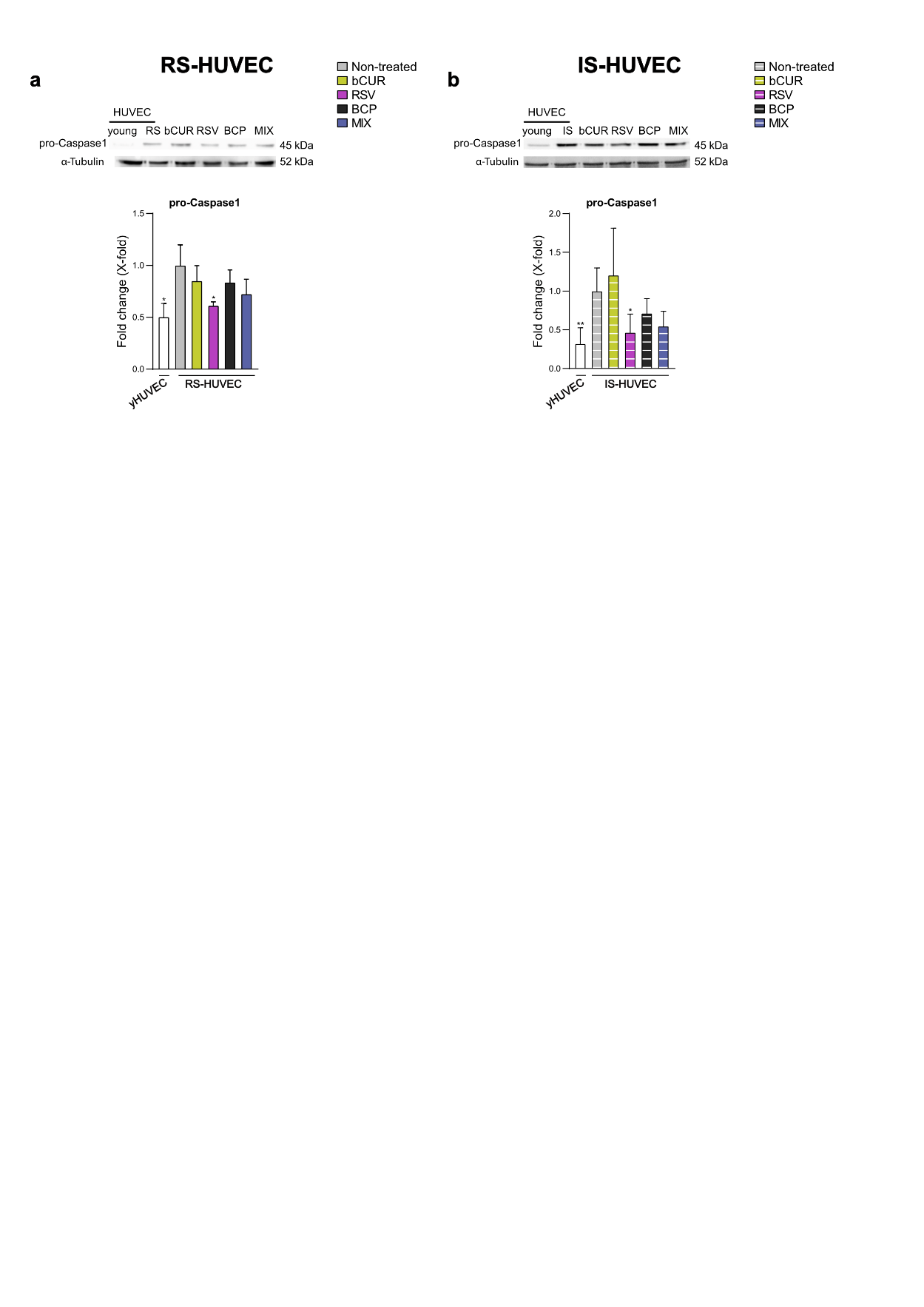


**Supplementary Figure 2 Pro-Caspase-1 protein level after treatment with bCUR, RSV and BCP and their combination in RS-(a) and IS-HUVECs (b)**. Pro-Caspase-1 protein level and densitometric analysis; (. Data are reported as fold change vs untreated senescent HUVECs All data were normalized using α-tubulin as internal control. Bands were quantified by ImageJ; histograms represent the mean of the protein expression detected in three different experiments ± SD. Paired t test *, p < 0.05 vs RS- and IS-HUVECs; ** p <0.01 vs RS- and IS-HUVECs. bCUR, bioCurcumin; RSV, Resveratrol; BCP, β‐caryophyllene; RS, replicative senescence; IS, induced senescence. RS-HUVECs data are depicted in the left panel (solid-coloured histograms); IS-HUVECs data are reported in the right panel (striped histograms).
